# Research Progress on the Role of Inflammasomes in Kidney Disease

**DOI:** 10.1155/2020/8032797

**Published:** 2020-01-29

**Authors:** Kun Chi, Xiaodong Geng, Chao Liu, GuangYan Cai, Quan Hong

**Affiliations:** Department of Nephrology, Chinese PLA General Hospital, Medical School of Chinese PLA, Chinese PLA Institute of Nephrology, State Key Laboratory of Kidney Diseases, National Clinical Research Center for Kidney Diseases, Beijing Key Laboratory of Kidney Diseases, 28 Fuxing Road, Beijing 100853, China

## Abstract

Inflammasomes are multimeric complexes composed of cytoplasmic sensors, apoptosis-associated speck-like protein containing a caspase activation and recruitment domain (ASC or PYCARD), and procaspase-1 and play roles in regulating caspase-dependent inflammation and cell death. Inflammasomes are assembled by sensing pathogen-associated molecular patterns (PAMPs) and damage-associated molecular patterns (DAMPs) and initiate inflammatory responses by activating caspase-1. Activated caspase-1 promotes the release of the inflammatory cytokines interleukin-1*β* (IL-1*β*) and IL-18 and eventually induces pyroptosis. Inflammasomes are closely related to kidney diseases. In particular, the NLRP3 (NACHT, LRR, and PYD domain-containing protein 3) inflammasome has been shown to cause acute and chronic kidney diseases by regulating canonical and noncanonical mechanisms of inflammation. Small-molecule inhibitors that target NLRP3 and other components of the inflammasome are potential options for the treatment of kidney-related diseases such as diabetic nephropathy. This article will focus on the research progress on inflammasomes and the key pathogenic roles of inflammasomes in the development and progression of kidney diseases and explore the potential of this intracellular inflammation to further prevent or block the development of the kidney disease.

## 1. Introduction

Since discovery, in the innate immune process, the role of inflammasomes cannot be ignored to endogenous and exogenous danger signals [1]. Inflammasomes are protein complexes that mainly contain a receptor protein, an adaptor protein, and downstream caspases, most notably caspase-1. When activated by an agonist, the receptor protein will then attract ASC and caspase-1 to assemble an inflammasome with a diameter up to micron size, thereby inducing self-cleavage and caspase-1 activation. On the one hand, activated caspase-1 will cause the release of IL-1*β*, IL-18, and other inflammatory factors; on the other hand, it promotes pyroptosis, which can help clear pathogens and damaged cells. Receptor proteins include NACHT, LRR, and PYD domain-containing protein 1 (NLRP1), NLRP3, and NLR family CARD domain-containing protein 4 (NLRC4), which belong to the NOD-like receptor family, as well as absent in melanoma-2 (AIM2) and pyrin. Diverse and specific receptor proteins can widely recognize exogenous (microbial molecules) and endogenous (danger signals) stimuli. The activation of inflammasomes can be regarded as the beginning of innate immune process, and it is also a sign of human resistance to infected tissues. Further studies have shown that noninfectious immune responses can also lead to the activation of inflammasomes [2–4]. Inflammasomes are involved in the occurrence and progression of various diseases in the human body including metabolic diseases (e.g., type 2 diabetes [5] and gouty arthritis [6]), malignant tumor [7], chronic arterial injury [8], and neurodegenerative disease [9–11]. Recent studies have shown that the formation and activation of inflammasomes occur in not only immune cells in the kidneys, such as activated macrophages, dendritic cells, and renal tissue cells (renal tubular epithelial cells and podocytes). These results fully show that inflammasomes play an important role in the development of renal diseases.

## 2. Canonical Inflammasomes

**Figure 1:**
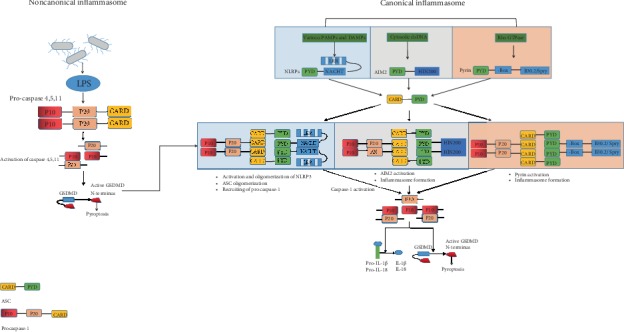
Canonical NLRPs can be activated by a variety of PAMPs and DAMPs, including influenza viruses, Listeria monocytogenes, extracellular ATP, hyaluronic acid, glucose, amyloid-*β*, silicon, and cholesterol crystals. These stimuli activate NLRP inflammasomes by triggering several common activation mechanisms, resulting in NLRP oligomerization. Active NLRPs interact with ASC to induce oligomerization, thereby forming protein complexes, further recruiting procaspase-1 to ASC to form inflammasomes, and leading to the cleavage of procaspase-1 into active caspase-1. Activated caspase-1 causes the cleavage of pro-IL-1*β* and pro-IL-18, leading to the maturation and secretion of various proinflammatory cytokines, including IL-1*β* and IL-18, inducing inflammatory responses. The cleavage of GSDMD and caspase-1 produces an N-terminal fragment (GSDMD-NT) that has affinity for phosphatidylinositol, phosphatidylserine, and cardiolipin. This fragment creates pores on the cell membrane, resulting in pyroptosis. The formation of pores also allows the extracellular release of active IL-1*β* and IL-18, thereby promoting an inflammatory response. After AIM2 and pyrin are activated by dsDNA and Rho GTPase, respectively, ASC and caspase-1 are also recruited to form canonical inflammasomes. Lysis of pro-IL-1*β*, pro-IL-18, and GSDMD results in the release of inflammatory factors and pyroptosis. Noncanonical inflammasomes activate caspase-11, caspase-4, and caspase-5 by directly binding to intracellular LPS and its component lipid A via their CARD domain. GSDMD is subsequently cleaved, and pyroptosis is induced. Activation of noncanonical inflammasomes may also lead to the assembly of canonical NLRP3 inflammasomes and induce the canonical inflammasome pathway, the mechanism of which may be related to mitochondrial ROS production and intracellular K+ efflux.

Canonical inflammasomes mainly include the NOD-like receptor (NLR) family, AIM2, and pyrin inflammasomes (Figure 1). In general, inflammasomes are polymer complexes composed of multiple pattern recognition receptors such as NLR and AIM2-like receptor (ALR), ASC, and caspase-1. NLRP1, NLRP3, ice protease-activating factor (IPAF) (also known as NLRC4), and AIM2 inflammasomes are examples of canonical inflammasomes. The activation of these types of inflammasomes is dependent on PRRs, which can only play a unique role in pathophysiology after detecting microbial pathogens or damage-related molecular patterns. Five PRR families have been identified: ALRs, NLRs, RIG-I-like receptors (RLRs), Toll-like receptors (TLRs), and C-type lectin receptors (CLRs). The PRRs have no transmembrane domain in the structure of inflammasomes, which exists in the cytoplasm. The role of inflammasomes and homologous proteins is to hydrolyze related proteins through the pyrin domain (PYD) and/or caspase activation and recruitment domain (card) and finally activate caspase-1, which further makes cytokines such as IL-1*β* and IL-18 matured and secreted in large quantities, as well as cleavage of gasdermin D (GSDMD), eventually causing pyroptosis [12].

NLRP3 inflammasomes are the most studied canonical inflammasome. NLRP3 is activated by sensing the presence of a variety of pathogens or danger signals and is involved in the occurrence and development of many major human diseases, such as atherosclerosis, Alzheimer's disease, inflammatory bowel disease, type 2 diabetes, and malignant tumors. NLRP3 inflammasomes are composed of NLRP3, a receptor protein containing a card structure that binds to the activated caspase-1 protein to regulate the intracellular expression of IL-1*β*. The structure of NLRP3 contains 9 C-terminal domains and a core region of nucleotide-binding fragments, rich in multiple leucine repeat (LLRs) and an amino terminal (N-terminal). [13, 14]. The NACHT domain has ATPase activity, and its regulation induces protein oligomerization required for the assembly of inflammasomes [15, 16]. Furthermore, it was confirmed that NLRP3 mutations occurred in many sites in the cells of patients with cryopyrin-associated cycle syndrome (CAPS), especially in the vicinity of ATP binding sites in Nacht domain [17]; therefore, new NLRP3 inhibitors (such as BAY 11-7082 and OLT1177) target this ATPase [18–20].

The activation of NLRP3 inflammasome is divided into two stages: priming/licensing and activation. PAMPs and/or DAMPs bind to TLRs and/or cytokine receptors to stimulate priming/licensing steps, and NF-*κ*B signal transduction and downstream gene regulation are usually involved; subsequently, inflammasome-associated genes and substrates were upregulated, such as pro-IL-1*β* [21]. This priming/licensing step also regulates the posttranslational modifications required for NLRP3 oligomerization, including deubiquitination, nitrosylation, and dephosphorylation [22, 23]. After the NLRP3 pathway is activated, the inflammasomes and caspase will then start to assemble and activate. Canonical NLRP3 inflammasome will be activated with the participation of various PAMPs and DAMPs, such as extracellular ATP, Listeria monocytogenes, influenza virus, hyaluronic acid, glucose, amyloid-*β*, silicon, and cholesterol crystals. These PAMPs and DAMPs activate NLRP3 inflammasome by triggering several common NLRP3 inflammasome activation mechanisms, such as K^+^ efflux from the cytoplasm [23] and mitochondrial events [24] and oxidative mitochondrial DNA [25] or ion efflux [23] due to lysosome degradation. After the inflammasomes become activated, they mediate the high secretion of many cytokines downstream of caspase-1 pathway, which makes GSDMD cleavage and pyrophosphorylation. Caspase-1 cleaves GSDMD, resulting in the release of the N-terminal domain of gasdermin D from the autoinhibitory C-terminal domain to the plasma membrane, forming 10-15 nm size pores in the plasma membrane, and eventually causing cell death [26].

## 3. Noncanonical Inflammasomes

Noncanonical functions of inflammasomes include the activated forms of other caspases, which are independent of caspase-1 (Figure 1). The noncanonical inflammasome pathway mainly activates caspase-4 and caspase-5 in humans and caspase-11 in mice [27–29]. In addition, inflammasome-mediated activation of caspase-8 is considered to be a noncanonical inflammasome pathway and may lead to IL-1*β* maturation [25, 30]. Inflammasomes can directly bind to intracellular lipopolysaccharide (LPS) and its component lipid A with the aid of card domain [31, 32] to activate caspase-11, caspase-4, and caspase-5. Subsequently, GSDMD is cleaved, inducing pyroptosis. However, the activation of these noncanonical inflammasomes may also lead to the assembly of canonical NLRP3 inflammasomes [30, 31], thereby inducing the canonical inflammasome pathway. The mechanisms underlying the induction of the canonical inflammasome pathway may be related to the mitochondrial ROS production and intracellular K^+^ efflux.

## 4. Inflammasomes and Kidney Disease

In recent years, studies have shown that inflammasomes play important roles in a variety of diseases, including autoimmune diseases, infections, and noninfectious diseases. Similarly, in kidney diseases, inflammasomes participate in the inflammatory reactions in kidneys, causing pathological lesions and kidney injury, indicating that inflammasomes play important roles in the occurrence and development of kidney diseases.

### 4.1. NLRP3 Inflammasome and Kidney Diseases

Numerous studies have confirmed that NLRP3 is the most typical inflammasome in the kidney, plays an important regulatory role in a variety of kidney diseases, and affects disease progression.

#### 4.1.1. Chronic Glomerulonephritis (GN)

Chronic glomerulonephritis will develop into uremia. During the course of the experimental nephrotoxic nephritis (NTN) animal model, it has been determined that the role of IL-1, tumor necrosis factor (TNF), and IL-1R is the cause of the decline of glomerular function [32]. High expression of key protein genes related to the formation of inflammasomes, such as renal dendritic cells, IL-1*β*, was detected in the cells of nephrotoxic nephritis animal model. However, the inflammatory process of renal tissue in NTN mice with NLRP3 and ASC gene expression blocked will be reversed [33].

Lichtnekert et al. observed that in a mouse model of antiglomerular basement membrane disease, IL-1R knockout mice had reduced levels of IL-1, which can protect mice from damage caused by necrotizing glomerulonephritis and crescentic glomerulonephritis. IL-18 deficiency did not result in glomerular protection, but tubular atrophy was alleviated [34]. The inflammasome pathway was studied in mice with targeted deletion of NLRP3, ASC, or caspase-1, and no difference in glomerular pathology was found. Deplano et al. conducted a study using a rat model of NTN combined with crescentic glomerulonephritis and found that P2X purinoceptor 7 (P2X7) receptor (P2X7R) antagonists inhibited the activation of NLRP3 and crescentic glomerular injury [35].

#### 4.1.2. Diabetic Nephropathy

The morbidity and mortality of diabetic nephropathy (DN) remain high; the main reason is ESRD. Genome-related studies have found that many types of inflammatory reactions in renal tissues of DN patients are activated, and many theories such as high glucose and activation of autoimmune mechanism have also been proposed [36].

Increasing data have indicated that inflammatory cell infiltration is very important in the pathogenesis of DN [5]. IL-18 and IL-1*β* secreted by immune cells and intrinsic glomerular cells (such as podocytes, endothelial cells, and mesangial cells) may promote the progression of DN [37, 38]. A preliminary study by Shahzad et al. showed that compared with those in nondiabetic mice, the expression levels of inflammasome molecules and proinflammatory cytokines in diabetic mice were upregulated [32]. After transplanting bone marrow from NLRP3- and caspase-1-deficient mice into db/db diabetic mice, the severity of kidney injury in diabetic mice was similar to that in the control group, and the activation of NLRP3 inflammasome derived from intrinsic renal cells aggravated DN. IL-1R antagonists and mitochondrial ROS inhibitors can be used as targeted therapy for DN by reducing NLRP3 inflammasome formation [32]. Mitochondrial ROS have been shown to activate NLRP3 inflammasome, further confirming the association between NLRP3 inflammasome activation and DN [39]. In addition, high-glucose treatment can induce the activation of nicotinamide adenine dinucleotide phosphate (NADPH) oxidase in mice, thereby triggering the activation of NLRP3 inflammasome in glomerular podocytes, subsequently causing podocyte injury. Downregulating the expression of thioredoxin-interacting protein (TXNIP) via small hairpin RNA (shRNA) and TXNIP inhibitors can block the activation of inflammasomes that can induce DN [40]. Therefore, inhibiting the expression of NLRP3 or caspase-1 can lead to inflammasome inactivation, which has a protective effect on renal tissues and may be a potential target for future DN treatment.

#### 4.1.3. Lupus Nephritis

In a mouse model of lupus nephritis, several experiments have confirmed that inflammasomes play key roles in the occurrence and development of the disease. Zhao et al. found that blocking P2X7R by inhibiting NLRP3 activation and subsequent caspase-1 activity reduced glomerular injury in MRL/lpr mice, thereby reducing serum anti-dsDNA antibody levels and IL-1*β* and IL-17 levels [41]. BAY 11-7082, a phosphorylated NF-*κ*B inhibitor (I*κ*B), reduced macrophage infiltration and inhibited the activation of NLRP3 inflammasome, thereby alleviating lupus nephritis in mice and improving the mouse survival rate [42]. However, this study analyzed only whole kidney lysates and did not clarify the cellular localization of inflammasome activity in the kidney. Therefore, the effect of circulating macrophages could not be ruled out. The observation and study of NZB/WF1 lupus nephritis mouse model showed that TLR7, TLR8, and TLR9 antagonists can effectively alleviate the progress of glomerular damage and interstitial inflammation while reducing the transcription levels of IL-1*β* and NLRP3 in the kidney [43].

#### 4.1.4. Crystalline Nephropathy

Crystalline nephropathy (heterogeneous nephropathy characterized by severe symptomatology from crystal embolization to kidney stones in the urethra) is also associated with canonical NLRP3 inflammasome. Calcium oxalate is the main component of kidney stones and is related to not only kidney stones but also acute kidney injury (AKI) and chronic kidney disease (CKD) [44]. Under the action of calcium oxalate crystal, NLRP3 inflammasomes are activated to activate the function of renal dendritic cells [45]. Inflammatory cells detected in various tissues of mice and human body can be found to contain a large number of cystine crystals in their lysosomes. It is concluded that these substances are the key to the activation of NLRP3 inflammasome and thus to promote the secretion of IL-1*β* [46]. Tubular cells can secrete Tamm-Horsfall protein, which makes nanoparticles appear in the diseased tubular tissue. These nanoparticles are an endogenous danger signal that will activate NLRP3 inflammasome after phagocytosis by macrophages [47]. Studies have shown that human monocyte phagocytes, such as dendritic cells and monocytes, can phagocytize urinary regulatory protein crystals in large quantities, release cathepsin, and activate inflammasomes through oxidative stress and potassium efflux pathway. [47].

#### 4.1.5. AKI

In ischemic reperfusion injury (IRI) [48], folic acid-induced nephropathy [49], cisplatin-induced AKI [50], rhabdomyolysis-induced AKI [51], and contrast-induced AKI [52] models, NLRP3-mediated activation of inflammasomes can be detected, and the progression of AKI is mediated through canonical and noncanonical inflammasome pathways. Compared with wild-type mice, NLRP3^−/−^ and ASC^−/−^ mice that received bilateral renal IRI reportedly exhibited a higher survival rate, improved renal function, and decreased neutrophil influx [53]. However, another study showed that the renal protective effect of NLRP3 deficiency was not associated with caspase-1 and ASC because after ASC and Csp1 knockout, the kidneys of IRI mice were not protected [54]. These findings indicate that the noncanonical inflammasome function of NLRP3 may play an important role in the pathogenesis of renal IRI.

The activation of canonical inflammasomes was also reported in an infection-induced AKI model. In an AKI model induced by sepsis caused by cecal ligation and puncture [55], the deletion of NLRP3 gene and the inhibition of caspase-1 will effectively block the inflammatory process and improve the renal function. In another study, the loss of caspase-1 reduced the mortality of endotoxin-induced AKI and hypotension-induced mouse models. [56].

#### 4.1.6. Hypertensive Nephropathy

Managing hypertension as well as other risk factors for kidney disease, such as elevated blood glucose levels and proteinuria, is critical to delaying the progression of ESRD. The expression of NLRP3 mRNA in renal biopsy specimens of patients with hypertensive or vascular nephrosclerosis is higher than that in normal kidneys, suggesting that NLRP3 plays a role in hypertension-related kidney disease [57]. Krishnan et al. indicated that activation of the NLRP3 inflammasome in the kidney is associated with salt-sensitive hypertension [58, 59]. Interestingly, in a hypertensive mouse model induced by angiotensin II, NLRP3 deficiency showed a protective effect on blood pressure, while the deletion of ASC did not [60]. ASC deficiency and IL-1R antagonism have blood pressure-lowering and renal anti-inflammatory effects [58, 61]. Activation of the NLRP3 inflammasome regulates Ang II-induced podocyte injury by inducing mitochondrial dysfunction, and systemic deletion of NLRP3 can significantly attenuate renal injury resulting from mitochondrial dysfunction [62]. The application of aldosterone can activate NLRP3 in podocytes through oxidative stress-related mechanisms. Inactivation of NLRP3 by siRNA can inhibit the NLRP3 activation and apoptosis induced by aldosterone and the loss of the podocyte-specific proteins nephrin and podocin. Additionally, systemic deletion of NLRP3 in mice remarkably ameliorate proteinuria and podocyte damage [63]. In a hypertensive mouse model, MCC950, an NLRP3 inhibitor, can significantly reduce blood pressure and fibrosis, alleviate kidney inflammation, and protect against renal dysfunction [59]. Current studies have shown that the investigation of inflammasome-independent functions is of great significance to discover the pathogenesis of hypertension [64].

### 4.2. Other Inflammasomes and Kidney Diseases

AIM2 inflammasomes, which are DNA-sensitive, have been reportedly associated with the pathogenesis of human hepatitis B virus-related glomerulonephritis [65] and mouse and human systemic lupus erythematosus [66]. AIM2 is constitutively expressed in glomerular podocytes and expressed at low levels in renal tubular epithelial cells in healthy individuals. Activation of AIM2 inflammasomes helps to recruit proinflammatory macrophages, leading to kidney damage and fibrosis, resulting in human CKD kidney injury in a mouse unilateral ureter obstruction (UUO) model [67]. The presence of nlrc4 inflammasomes can be detected in both human and mouse DKD progression [68]. Compared with the wild-type diabetic mice, the blood glucose level of the model with low expression of nlrc4 decreased significantly, and the glomerular injury was reversed to some extent. Molecular biology studies have shown that nlrc5 is a transcriptional activator of class I gene of histocompatibility complex (MHC) [69]. In the AKI mouse model induced by IRI or cisplatin, the overexpression of nlrc5 can be detected, while the expression of carcinoembryonic antigen-related cell adhesion molecule is inhibited, ERK1/2 and Akt signaling pathway are activated significantly, and the degree of renal damage is particularly serious [70]. Bone marrow chimerism-related experiments also showed the key role of nlrc5 in AKI renal tubular injury and renal failure animal model [70]. NLRX1 is located in mitochondria, and its anti-inflammatory effect is very obvious [71, 72]. In renal IRI, NLRX1 can inhibit the oxidative phosphorylation process, protect the integrity of cell membrane, and play a role in protecting renal parenchymal cells. [73].

## 5. Therapeutic Prospects of Targeting Inflammasomes in Renal Diseases

To date, immunomodulatory strategies targeting inflammasomes have mainly focused on downstream effective substances, IL-1 and caspase-1. Currently, there are several studies on targeted therapeutics for upstream and downstream effective substances of inflammasomes in kidney diseases; however, their clinical efficacy still needs to be validated.

Diarylsulfonylurea compounds can inhibit LPS- and ATP-induced IL-1*β* processing and release in human monocytes and murine macrophages [74], and MCC950 is a specific inhibitor of NLRP3 activation [75]. In a mouse model of crystalline nephropathy caused by a high-adenine diet [76], MCC950 significantly inhibited the production of IL-1*β* and IL-18 caused by the activation of NLRP3 inflammasome. Significant inhibitory effects of MCC950 on IL-1*β* maturation in bone marrow cells have been shown using an *in vivo* bioluminescence imaging system. In a rat model of DN, BAY 11-7082 inhibited NLRP3 ATPase activity, which is required for inflammasome activation [18], normalized the levels of proinflammatory cytokines and oxidative stress, and played a role in renal protection [77].

The protective effect of caspase-1 inhibitor on renal function has been widely confirmed. Some studies have shown that the effect of AC-YVAD-CMK (caspase-1 inhibitor) on the recovery of renal function in AKI model of rodents is very obvious [55, 78]. VX-765 (highly selective caspase-1 inhibitor) can effectively improve the renal fibrosis in mice with nephropathy [79].

The catalytic domain of caspases will directly bind to GSDMD and its cleavage site peptide fltd [80]. Ac-FLTD-cmk, a GSDMD-derived peptide inhibitor, can inhibit the cleavage of GSDMD by binding directly to the core catalytic domain of caspase-1, caspase-4, caspase-5, and caspase-11. Without affecting cell apoptosis, it can reduce the thermoptosis and effector function of typical and atypical inflammasomes. BAY 11-7082 (inhibitors of NF-*κ*B and NLRP3) can covalently modify the active cysteine in GSDMD and inhibit the pore formation induced by GSDMD [81].

Anakinra is an artificial recombinant IL-1 receptor antagonist, which has a significant therapeutic effect on tumor necrosis factor receptor-associated cycle syndrome (traps) [82], gouty arthritis [83], and diabetes [84]. The IL-1 trap rilonacept binds and neutralizes IL-1 and controls the symptoms of CAPS and gouty arthritis [85].

Although agents targeting inflammasomes have great potential in disease treatment, there are also potential limitations of inflammasome inhibition in the clinic. MCC950 is a promising drug, and numerous targeted studies have been carried out, showing a broad prospect of treatments. However, related studies can prove only that short-term animal experiments can alleviate diseases under laboratory conditions, but the long-term effects of MCC950 on related animals have not been studied. In addition, MCC950 has some immunosuppressive side effects, such as susceptibility to infection [86]. BAY 11-7082 is a specific pharmacologic NF-*κ*B inhibitor with obvious cytotoxicity, which leads to increasing cell death as the concentration increases [87]. Some studies have shown that BAY 11-7082 impairs alveolar epithelial barrier function and causes unavoidable toxicity in cells and cultured brain tissue [88, 89]. In the application of BAY 11-7085 system, toxic reactions were detected, so the local application of NF-*κ*B inhibitor was paid more attention by many scholars. Local reactions at the injection site are the most common side effects of anakinra. In addition, the risk of serious infection is reported to increase [90].

## 6. Summary and Outlook

Over the past 10 years, substantial progress has been made in the study of inflammasomes. Increasing evidence has confirmed the association between the canonical and noncanonical functions of inflammasomes and kidney diseases, but many issues remain to be addressed, such as the effects of noncanonical caspase-11, caspase-4, and caspase-5 inflammasomes and other NLR genes; the role of pyroptosis; the activation of noncanonical inflammasomes in the kidney; and the function of gasdermin. Studying the mechanisms of these functions can help to understand the association between inflammation and kidney diseases. The ongoing development of therapy involving targeting inflammasomes and their downstream effectors, including caspase-1 and IL-1*β*, has been shown to be effective *in vitro* and in many mouse models. However, converting these experimental data into clinical applications still requires further exploration. These studies may advance the treatment of kidney diseases such as CKD and AKI.

## References

[1] He W. T., Wan H., Hu L. (2015). Gasdermin D is an executor of pyroptosis and required for interleukin-1*β* secretion. *Cell Research*.

[2] Dostert C., Petrilli V., Van Bruggen R., Steele C., Mossman B. T., Tschopp J. (2008). Innate immune activation through Nalp3 inflammasome sensing of asbestos and silica. *Science*.

[3] Duewell P., Kono H., Rayner K. J. (2010). NLRP3 inflammasomes are required for atherogenesis and activated by cholesterol crystals. *Nature*.

[4] Halle A., Hornung V., Petzold G. C. (2008). The NALP3 inflammasome is involved in the innate immune response to amyloid-*β*. *Nature Immunology*.

[5] Awad A. S., Kinsey G. R., Khutsishvili K., Gao T., Bolton W. K., Okusa M. D. (2011). Monocyte/macrophage chemokine receptor CCR2 mediates diabetic renal injury. *American Journal of Physiology-Renal Physiology*.

[6] Martinon F., Petrilli V., Mayor A., Tardivel A., Tschopp J. (2006). Gout-associated uric acid crystals activate the NALP3 inflammasome. *Nature*.

[7] Karasawa T., Takahashi M. (2017). Role of NLRP3 inflammasomes in atherosclerosis. *Journal of Atherosclerosis and Thrombosis*.

[8] Freeman L. C., Ting J. P.-Y. (2016). The pathogenic role of the inflammasome in neurodegenerative diseases,. *Journal of Neurochemistry*.

[9] Zhiyu W., Wang N., Wang Q. (2016). The inflammasome: an emerging therapeutic oncotarget for cancer prevention. *Oncotarget*.

[10] Man S. M., Zhu Q., Zhu L. (2015). Critical role for the DNA sensor AIM2 in stem cell proliferation and cancer. *Cell*.

[11] Aubert D. F., Xu H., Yang J. (2016). A _Burkholderia_ Type VI Effector Deamidates Rho GTPases to Activate the Pyrin Inflammasome and Trigger Inflammation. *Cell Host Microbe*.

[12] Rathinam V. A. K., Chan F. K.-M. (2018). Inflammasome, inflammation, and tissue homeostasis. *Trends in Molecular Medicine*.

[13] Place D. E., Kanneganti T. D. (2018). Recent advances in inflammasome biology. *Current Opinion in Immunology*.

[14] Schroder K., Tschopp J. (2010). The inflammasomes. *Cell*.

[15] Duncan J. A., Bergstralh D. T., Wang Y. (2007). Cryopyrin/NALP3 binds ATP/dATP, is an ATPase, and requires ATP binding to mediate inflammatory signaling. *Proceedings of the National Academy of Sciences*.

[16] Latz E., Xiao T. S., Stutz A. (2013). Activation and regulation of the inflammasomes. *Nature Reviews Immunology*.

[17] MacDonald J. A., Wijekoon C. P., Liao K.-C., Muruve D. A. (2013). Biochemical and structural aspects of the ATP-binding domain in inflammasome-forming human NLRP proteins. *IUBMB Life*.

[18] Juliana C., Fernandes-Alnemri T., Wu J. (2010). Anti-inflammatory compounds parthenolide and Bay 11-7082 are direct inhibitors of the inflammasome. *Journal of Biological Chemistry*.

[19] Marchetti C., Swartzwelter B., Gamboni F. (2018). OLT1177, a *β*-sulfonyl nitrile compound, safe in humans, inhibits the NLRP3 inflammasome and reverses the metabolic cost of inflammation. *Proceedings of the National Academy of Sciences*.

[20] Mastrocola R., Penna C., Tullio F. (2016). Pharmacological inhibition of NLRP3 inflammasome attenuates myocardial ischemia/reperfusion injury by activation of RISK and mitochondrial pathways. *Oxidative Medicine and Cellular Longevity*.

[21] Rathinam V. A. K., Vanaja S. K., Fitzgerald K. A. (2012). Regulation of inflammasome signaling. *Nature Immunology*.

[22] Mishra B. B., Rathinam V. A. K., Martens G. W. (2013). Nitric oxide controls the immunopathology of tuberculosis by inhibiting NLRP3 inflammasome-dependent processing of IL-1*β*. *Nature Immunology*.

[23] Munoz-Planillo R., Kuffa P., Martinez-Colon G., Smith B. L., Rajendiran T. M., Nunez G. (2013). K^+^ Efflux Is the Common Trigger of NLRP3 Inflammasome Activation by Bacterial Toxins and Particulate Matter. *Immunity*.

[24] Zhou R., Yazdi A. S., Menu P., Tschopp J. (2011). A role for mitochondria in NLRP3 inflammasome activation. *Nature*.

[25] Zhong Z., Liang S., Sanchez-Lopez E. (2018). New mitochondrial DNA synthesis enables NLRP3 inflammasome activation. *Nature*.

[26] Shi J., Zhao Y., Wang K. (2015). Cleavage of GSDMD by inflammatory caspases determines pyroptotic cell death. *Nature*.

[27] Kayagaki N., Warming S., Lamkanfi M. (2011). Non-canonical inflammasome activation targets caspase-11. *Nature*.

[28] Schmid-Burgk J. L., Gaidt M. M., Schmidt T., Ebert T. S., Bartok E., Hornung V. (2015). Caspase-4 mediates non-canonical activation of the NLRP3 inflammasome in human myeloid cells. *European Journal of Immunology*.

[29] Shi J., Zhao Y., Wang Y. (2014). Inflammatory caspases are innate immune receptors for intracellular LPS. *Nature*.

[30] Ruhl S., Broz P. (2015). Caspase-11 activates a canonical NLRP3 inflammasome by promoting K(+) efflux. *European Journal of Immunology*.

[31] Platnich J. M., Chung H., Lau A. (2018). Shiga Toxin/Lipopolysaccharide Activates Caspase-4 and Gasdermin D to Trigger Mitochondrial Reactive Oxygen Species Upstream of the NLRP3 Inflammasome. *Cell Reports*.

[32] Shahzad K., Bock F., Dong W. (2015). Nlrp3-inflammasome activation in non-myeloid-derived cells aggravates diabetic nephropathy. *Kidney International*.

[33] Andersen K., Eltrich N., Lichtnekert J., Anders H. J., Vielhauer V. (2014). The NLRP3/ASC inflammasome promotes T-cell-dependent immune complex glomerulonephritis by canonical and noncanonical mechanisms. *Kidney International*.

[34] Lichtnekert J., Kulkarni O. P., Mulay S. R. (2011). Anti-GBM glomerulonephritis involves IL-1 but is independent of NLRP3/ASC inflammasome-mediated activation of caspase-1. *PLoS One*.

[35] Deplano S., Cook H. T., Russell R. (2013). P2X7 receptor-mediated Nlrp3-inflammasome activation is a genetic determinant of macrophage-dependent crescentic glomerulonephritis. *Journal of Leukocyte Biology*.

[36] Petrilli V., Papin S., Dostert C., Mayor A., Martinon F., Tschopp J. (2007). Activation of the NALP3 inflammasome is triggered by low intracellular potassium concentration. *Cell Death & Differentiation*.

[37] Segelmark M., Hellmark T. (2010). Autoimmune kidney diseases. *Autoimmunity Reviews*.

[38] Terkeltaub R., Sundy J. S., Schumacher H. R. (2009). The interleukin 1 inhibitor rilonacept in treatment of chronic gouty arthritis: results of a placebo-controlled, monosequence crossover, non-randomised, single-blind pilot study. *Annals of the Rheumatic Diseases*.

[39] Han Y., Xu X., Tang C. (2018). Reactive oxygen species promote tubular injury in diabetic nephropathy: the role of the mitochondrial ros-txnip-nlrp3 biological axis. *Redox Biology*.

[40] Gao P., He F. F., Tang H. (2015). NADPH Oxidase-Induced NALP3 Inflammasome Activation Is Driven by Thioredoxin- Interacting Protein Which Contributes to Podocyte Injury in Hyperglycemia. *Journal of Diabetes Research*.

[41] Zhao J., Wang H., Dai C. (2013). P2X7 blockade attenuates murine lupus nephritis by inhibiting activation of the NLRP3/ASC/caspase 1 pathway. *Arthritis and Rheumatism*.

[42] Zhao J., Zhang H., Huang Y. (2013). Bay11-7082 attenuates murine lupus nephritis via inhibiting NLRP3 inflammasome and NF-*κ*B activation. *International Immunopharmacology*.

[43] Zhu F. G., Jiang W., Bhagat L. (2013). A novel antagonist of Toll-like receptors 7, 8 and 9 suppresses lupus disease-associated parameters in NZBW/F1 mice. *Autoimmunity*.

[44] Mulay S. R., Anders H. J. (2017). Crystal nephropathies: mechanisms of crystal-induced kidney injury. *Nature Reviews Nephrology*.

[45] Mulay S. R., Kulkarni O. P., Rupanagudi K. V. (2013). Calcium oxalate crystals induce renal inflammation by NLRP3-mediated IL-1*β* secretion. *The Journal of Clinical Investigation*.

[46] Prencipe G., Caiello I., Cherqui S. (2014). Inflammasome activation by cystine crystals: implications for the pathogenesis of cystinosis. *Journal of the American Society of Nephrology*.

[47] Darisipudi M. N., Thomasova D., Mulay S. R. (2012). Uromodulin triggers IL-1*β*–Dependent innate Immunityviathe NLRP3 inflammasome. *Journal of the American Society of Nephrology*.

[48] Tang T. T., Lv L. L., Pan M. M. (2018). Hydroxychloroquine attenuates renal ischemia/reperfusion injury by inhibiting cathepsin mediated NLRP3 inflammasome activation. *Cell Death and Disease*.

[49] Subramanian N., Natarajan K., Clatworthy M. R., Wang Z., Germain R. N. (2013). The adaptor MAVS promotes NLRP3 mitochondrial localization and inflammasome activation. *Cell*.

[50] Kim H.-J., Lee D. W., Ravichandran K. (2013). NLRP3 inflammasome knockout mice are protected against ischemic but not cisplatin-induced acute kidney injury. *Journal of Pharmacology and Experimental Therapeutics*.

[51] Komada T., Usui F., Kawashima A. (2015). Role of NLRP3 inflammasomes for rhabdomyolysis-induced acute kidney injury. *Scientific Reports*.

[52] Lau A., Chung H., Komada T. (2018). Renal immune surveillance and dipeptidase-1 contribute to contrast-induced acute kidney injury. *Journal of Clinical Investigation*.

[53] Iyer S. S., Pulskens W. P., Sadler J. J. (2009). Necrotic cells trigger a sterile inflammatory response through the Nlrp3 inflammasome. *Proceedings of the National Academy of Sciences*.

[54] Shigeoka A. A., Mueller J. L., Kambo A. (2010). An inflammasome-independent role for epithelial-expressed Nlrp3 in renal ischemia-reperfusion injury. *The Journal of Immunology*.

[55] Cao Y., Fei D., Chen M. (2015). Role of the nucleotide-binding domain-like receptor protein 3 inflammasome in acute kidney injury. *FEBS Journal*.

[56] Wang W., Faubel S., Ljubanovic D. (2005). Endotoxemic acute renal failure is attenuated in caspase-1-deficient mice. *American Journal of Physiology-Renal Physiology*.

[57] Vilaysane A., Chun J., Seamone M. E. (2010). The NLRP3 inflammasome promotes renal inflammation and contributes to CKD. *Journal of the American Society of Nephrology*.

[58] Krishnan S. M., Dowling J. K., Ling Y. H. (2016). Inflammasome activity is essential for one kidney/deoxycorticosterone acetate/salt-induced hypertension in mice. *British Journal of Pharmacology*.

[59] Krishnan S. M., Ling Y. H., Huuskes B. M. (2019). Pharmacological inhibition of the NLRP3 inflammasome reduces blood pressure, renal damage, and dysfunction in salt-sensitive hypertension. *Cardiovascular Research*.

[60] Shirasuna K., Karasawa T., Usui F. (2015). NLRP3 deficiency improves angiotensin II-induced hypertension but not fetal growth restriction during pregnancy. *Endocrinology*.

[61] Ling Y. H., Krishnan S. M., Chan C. T. (2017). Anakinra reduces blood pressure and renal fibrosis in one kidney/DOCA/salt-induced hypertension. *Pharmacological Research*.

[62] Zhao M., Bai M., Ding G. (2018). Angiotensin II stimulates the NLRP3 inflammasome to induce podocyte injury and mitochondrial dysfunction. *Kidney Disease*.

[63] Bai M., Chen Y., Zhao M. (2017). NLRP3 inflammasome activation contributes to aldosterone-induced podocyte injury. *American Journal of Physiology-Renal Physiology*.

[64] Kim Y. G., Kim S.-M., Kim K.-P., Lee S.-H., Moon J.-Y. (2019). The role of inflammasome-dependent and inflammasome-independent NLRP3 in the kidney. *Cells*.

[65] Zhen J., Le Zhang J. P., Ma S. (2014). AIM2 Mediates Inflammation-Associated Renal Damage in Hepatitis B Virus- Associated Glomerulonephritis by Regulating Caspase-1, IL-1*β*, and IL-18. *Mediators of Inflammation*.

[66] Zhang W., Cai Y., Xu W., Yin Z., Gao X., Xiong S. (2013). AIM2 facilitates the apoptotic DNA-induced systemic lupus erythematosus via arbitrating macrophage functional maturation. *Journal of Clinical Immunology*.

[67] Komada T., Chung H., Lau A. (2018). Macrophage uptake of necrotic cell DNA activates the AIM2 inflammasome to regulate a proinflammatory phenotype in CKD. *Journal of the American Society of Nephrology*.

[68] Yuan F., Kolb R., Pandey G. (2016). Involvement of the NLRC4-inflammasome in diabetic nephropathy. *PLoS One*.

[69] Meissner T. B., Li A., Biswas A. (2010). NLR family member NLRC5 is a transcriptional regulator of MHC class I genes. *Proceedings of the National Academy of Sciences*.

[70] Li Q., Wang Z., Zhang Y. (2018). NLRC5 deficiency protects against acute kidney injury in mice by mediating carcinoembryonic antigen-related cell adhesion molecule 1 signaling. *Kidney International*.

[71] Moore C. B., Bergstralh D. T., Duncan J. A. (2008). NLRX1 is a regulator of mitochondrial antiviral immunity. *Nature*.

[72] Arnoult D., Soares F., Tattoli I., Castanier C., Philpott D. J., Girardin S. E. (2009). An N-terminal addressing sequence targets NLRX1 to the mitochondrial matrix. *Journal of Cell Science*.

[73] Stokman G., Kors L., Bakker P. J. (2017). NLRX1 dampens oxidative stress and apoptosis in tissue injury via control of mitochondrial activity. *The Journal of Experimental Medicine*.

[74] Perregaux D. G., McNiff P., Laliberte R. (2001). Identification and characterization of a novel class of interleukin-1 post-translational processing inhibitors. *Journal of Pharmacology and Experimental Therapeutics*.

[75] Coll R. C., Robertson A. A. B., Chae J. J. (2015). A small-molecule inhibitor of the NLRP3 inflammasome for the treatment of inflammatory diseases. *Nature Medicine*.

[76] Ludwig-Portugall I., Bartok E., Dhana E. (2016). An NLRP3-specific inflammasome inhibitor attenuates crystal-induced kidney fibrosis in mice. *Kidney International*.

[77] Kolati S. R., Kasala E. R., Bodduluru L. N. (2015). BAY 11-7082 ameliorates diabetic nephropathy by attenuating hyperglycemia- mediated oxidative stress and renal inflammation via NF-*κ*B pathway. *Environmental Toxicology and Pharmacology*.

[78] Homsi E., Janino P., de Faria J. B. L. (2006). Role of caspases on cell death, inflammation, and cell cycle in glycerol- induced acute renal failure. *Kidney International*.

[79] Bialer M., Johannessen S. I., Levy R. H., Perucca E., Tomson T., White H. S. (2013). Progress report on new antiepileptic drugs: a summary of the Eleventh Eilat Conference (EILAT XI). *Epilepsy Research*.

[80] Yang J., Liu Z., Wang C. (2018). Mechanism of gasdermin D recognition by inflammatory caspases and their inhibition by a gasdermin D-derived peptide inhibitor. *Proceedings of the National Academy of Sciences*.

[81] Hu J. J., Liu X., Zhao J. (2018). Identification of pyroptosis inhibitors that target a reactive cysteine in gasdermin D. *bioRxiv*.

[82] Chang C., Bachove I. (2014). Anakinra and related drugs targeting interleukin-1 in the treatment of cryopyrin-associated periodic syndromes. *Open Access Rheumatology: Research and Reviews*.

[83] Ottaviani S., Molto A., Ea H. K. (2013). Efficacy of anakinra in gouty arthritis: a retrospective study of 40 cases. *Arthritis Research & Therapy*.

[84] Malozowski S., Sahlroot J. T. (2007). Interleukin-1-receptor antagonist in type 2 diabetes mellitus. *New England Journal of Medicine*.

[85] Sundy J. S., Schumacher H. R., Kivitz A. (2014). Rilonacept for gout flare prevention in patients receiving uric acid-lowering therapy: results of RESURGE, a phase III, international safety study. *The Journal of Rheumatology*.

[86] Dinarello C. A., van der Meer J. W. M. (2013). Treating inflammation by blocking interleukin-1 in humans. *Seminars in Immunology*.

[87] Tong H. B., Zou C. L., Qin S. Y. (2018). Prostate cancer tends to metastasize in the bone-mimicking microenvironment via activating NF-*κ*B signaling. *Journal Biomedical Research*.

[88] Nandhu M. S., Kwiatkowska A., Bhaskaran V., Hayes J., Hu B., Viapiano M. S. (2017). Tumor-derived fibulin-3 activates pro-invasive NF-*κ*B signaling in glioblastoma cells and their microenvironment. *Oncogene*.

[89] Li X., Du W., Ma F. X., Feng X., Bayard F., Han Z. C. (2015). High concentrations of TNF-*α* induce cell death during interactions between human umbilical cord mesenchymal stem cells and peripheral blood mononuclear cells. *PLoS One*.

[90] Cohen S. B., Moreland L. W., Cush J. J. (2004). A multicentre, double blind, randomised, placebo controlled trial of anakinra (Kineret), a recombinant interleukin 1 receptor antagonist, in patients with rheumatoid arthritis treated with background methotrexate. *Annals of the Rheumatic Diseases*.

